# EP4 as a Therapeutic Target for Aggressive Human Breast Cancer

**DOI:** 10.3390/ijms19041019

**Published:** 2018-03-29

**Authors:** Mousumi Majumder, Pinki Nandi, Ahmed Omar, Kingsley Chukwunonso Ugwuagbo, Peeyush K. Lala

**Affiliations:** 1 Department of Biology, Brandon University, Brandon, MB R7A6A9, Canada; majumderm@brandonu.ca (M.M.); ismailao09@brandonu.ca (A.O.); ugwuagkc97@brandonu.ca (K.C.U.); 2 Department of Anatomy and Cell Biology, Schulich School of Medicine and Dentistry, University of Western Ontario, London, ON N6A5C1, Canada; pnandi@uwo.ca; 3 Department of Oncology, Schulich School of Medicine and Dentistry, University of Western Ontario, London, ON N6A5C1, Canada

**Keywords:** COX-2, breast cancer, PGE2, EP receptors, stem-like cells, metastasis, angiogenesis, lymphangiogenesis, microRNAs, triple-negative breast cancer

## Abstract

G-protein-coupled receptors (GPCRs, also called seven-transmembrane or heptahelical receptors) are a superfamily of cell surface receptor proteins that bind to many extracellular ligands and transmit signals to an intracellular guanine nucleotide-binding protein (G-protein). When a ligand binds, the receptor activates the attached G-protein by causing the exchange of Guanosine-5′-triphosphate (GTP) for guanosine diphosphate (GDP). They play a major role in many physiological functions, as well as in the pathology of many diseases, including cancer progression and metastasis. Only a few GPCR members have been exploited as targets for developing drugs with therapeutic benefit in cancer. Present review briefly summarizes the signaling pathways utilized by the EP (prostaglandin E receptor) family of GPCR, their physiological and pathological roles in carcinogenesis, with special emphasis on the roles of EP4 in breast cancer progression. We make a case for EP4 as a promising newer therapeutic target for treating breast cancer. We show that an aberrant over-expression of cyclooxygenase (COX)-2, which is an inflammation-associated enzyme, occurring in 40–50% of breast cancer patients leads to tumor progression and metastasis due to multiple cellular events resulting from an increased prostaglandin (PG) E2 production in the tumor milieu. They include inactivation of host anti-tumor immune cells, such as Natural Killer (NK) and T cells, increased immuno-suppressor function of tumor-associated macrophages, promotion of tumor cell migration, invasiveness and tumor-associated angiogenesis, due to upregulation of multiple angiogenic factors including Vascular Endothelial Growth Factor (VEGF)-A, increased lymphangiogenesis (due to upregulation of VEGF-C/D), and a stimulation of stem-like cell (SLC) phenotype in cancer cells. All of these events were primarily mediated by activation of the Prostaglandin (PG) E receptor EP4 on tumor or host cells. We show that selective EP4 antagonists (EP4A) could mitigate all of these events tested with cells in vitro as well as in vivo in syngeneic COX-2 expressing mammary cancer bearing mice or immune-deficient mice bearing COX-2 over-expressing human breast cancer xenografts. We suggest that EP4A can avoid thrombo-embolic side effects of long term use of COX-2 inhibitors by sparing cardio-protective roles of PGI2 via IP receptor activation or PGE2 via EP3 receptor activation. Furthermore, we identified two COX-2/EP4 induced oncogenic and SLC-stimulating microRNAs—miR526b and miR655, one of which (miR655) appears to be a potential blood biomarker in breast cancer patients for monitoring SLC-ablative therapies, such as with EP4A. We suggest that EP4A will likely produce the highest benefit in aggressive breast cancers, such as COX-2 expressing triple-negative breast cancers, when combined with other newer agents, such as inhibitors of programmed cell death (PD)-1 or PD-L1.

## 1. Introduction

G-protein coupled receptors (GPCRs) are a superfamily of receptors that transduce signals by their coupling with guanine nucleotide-binding proteins (G-proteins). They include about 900 members, some with known ligands, others that were identified as orphan receptors. A diverse set of ligands, including peptide hormones, neurotransmitters, and odor molecules bind to GPCRs. They represent the most notable family of validated pharmacological targets in a variety of diseases, including cancer. Numerous GPCRs, such as receptors for chemokines, thrombin, lysophosphatidic acid (LPA), gastrin-releasing peptide, angiotensin, the sphingosine 1-phosphate, endothelin, and prostaglandins have been reported to play a key role in cancer progression and metastasis reviewed in [1,2]. The present article will focus on prostaglandin E receptor EP4 as a therapeutic target in aggressive breast cancer, including triple-negative breast cancer. 

## 2. Breast Cancer: Needs to Identify Novel Therapeutic Targets

Breast cancer accounts for the most frequent cancer in the female globally. It represents the second highest cause of cancer-related mortality in the western hemisphere due to the resistance of some 25–30% of the patients to currently practiced therapies such as surgery, radiotherapy, chemotherapy, hormone therapy, and Human Epidermal Growth Factor Receptor (HER) 2-targeted drugs, necessitating the search for newer therapy targets. Recent advances in cancer genomics have formed the basis of “Personalized medicine” in identifying therapeutic target(s) appropriate for the individual patient [3]. Genomic profiling of breast cancer by gene micro-array has recently been used to predict therapeutic outcome, which forms the basis for numerous commercially developed assays for use in the clinic reviewed in [4,5]. The remarkable advent of current high-throughput technologies in combination with improved knowledge of the molecular basis of malignancy provides a solid base for identifying novel molecular targets. As reviewed below, we show that 40–50% of breast cancer patients, including a most aggressive subset of patients identified as ER-/PR-/HER-2- or “triple-negative breast cancer (TNBC)” reveal an upregulation of the inflammation-associated enzyme cyclooxygenase (COX)-2, which drives tumor progression and metastasis, and that prostaglandin E receptor EP4, a GPCR family member, presents as a promising newer therapeutic target in these patients.

## 3. Cyclo-Oxygenase Pathway

**Figure 1:**
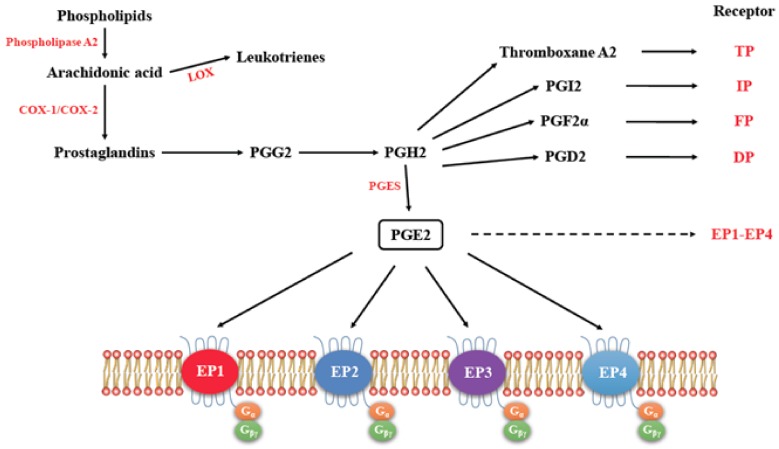
The pathway for the synthesis of prostaglandins, their respective receptors and signaling. (Adapted with kind permission from Markovič, T.; et al. 2017; reference [9]). Arachidonic acid acts as the substrate for cyclooxygenase (COX)-1 and COX-2 to produce Prostaglandins PGE2, Thromboxane A2, PGI2, PGF2α, and PGD2, all of which exert functions by binding to their respective receptors. EP1-EP4 (dashed arrow) receptors are further detailed for their G protein coupling.

Molecular cascade in the COX pathway has been adequately reviewed [6,7]. Briefly, COX family of enzymes includes three members: COX-1, COX-2, and COX-3. COX-3 is an isoform of COX-1 produced by alternative splicing of *COX-1* or *PTGS-1* gene, and is not present in the human. Most somatic cells constitutively express COX-1, and a small minority of cells (of the reproductive and immune systems) constitutively expresses COX-2. Cell membrane phospholipids, under the influence of phospholipase A2 (PLA2) produce Arachidonic acid, which acts as the substrate for lipoxygenases (LOX) to produce leukotrienes and cyclooxygenases (COX) to produce prostaglandins PGE2, Thromboxane A2, PGI2, PGF2α, and PGD2, all of which exert physiological functions by binding to their respective receptors (EP family for PGE2, TP for Thromboxane A2, IP for PGI2, FP for PGF2α, and DP for PGD2). PGE2 is the most abundant eicosanoid that is produced by the action of PGE synthase (PGES) enzymes on PGG2 downstream of COX (Figure 1). Secreted PGE2 is a short-lived molecule, which is quickly catabolized to the inactive 15-keto-PGE by the enzyme 15-hydroxyprostaglandin dehydrogenase (15-PGDH, also known as HPGD). PGE2 acts locally in an autocrine or paracrine manner through its four cognate G-protein coupled receptors EP1 to EP4. Under physiological conditions, PGE2 mediates many biological functions, such as the regulation of immune responses, blood pressure, gastrointestinal integrity, and fertility. Deregulated PGE2 synthesis or degradation is associated with many pathological conditions, like chronic inflammation, Alzheimer’s disease, and tumorigenesis. COX-2 is expressed constitutively only in a small minority of cells, such as macrophages and some cells in the reproductive organs. Typically, it is an inflammation-associated enzyme induced by inflammatory cytokines, mitogens, and certain carcinogens. PGE2 production via COX-1 pathway occurs steadily at low local concentrations. In contrast, COX-2-mediated PGE2 production during inflammation occurs at high local concentrations and stops after the withdrawal of the inflammatory stimulus. However, aberrant COX-2 activity that occurs in many epithelial cancers, including breast cancer, leads to persistent PGE2 production [7,8].

### EP Receptors

**Figure 2:**
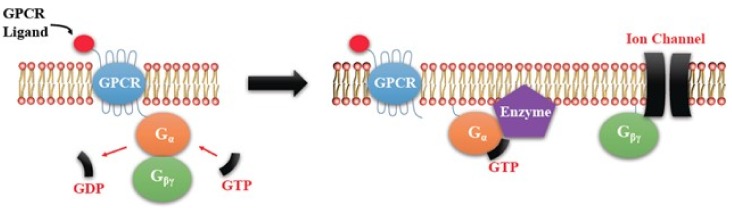
Heterotrimeric G-protein activation and inactivation cycle. The activation occurs by conversion of G-protein alpha (Gα)-coupled guanosine diphosphate (GDP) to Guanosine-5′-triphosphate (GTP). The activated G-protein then dissociates into an α and a β/γ complex. GTP bound Gα is active. Intrinsic GTPase activity leads to the inactivation of the G-Protein. GDP bound Gα re-associates with a β/γ complex to form the inactive G-protein that can again associate with a receptor.

**Figure 3:**
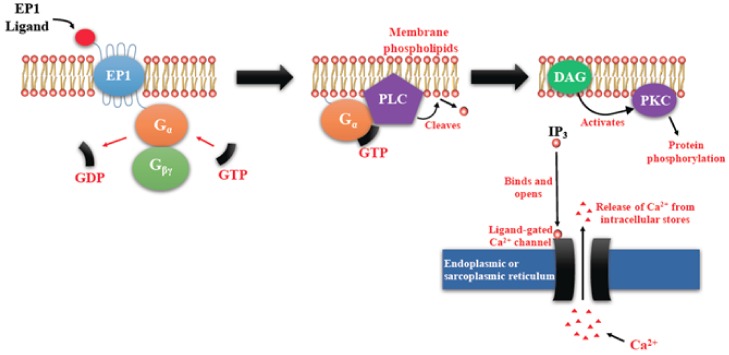
Prostaglandin E receptors (EP1)-mediated signaling events. EP1 couples with G_q_, activating PLC that cleaves PIP2, to generate second messengers, IP_3_, and diacylglycerol (DAG). IP_3_ binds to and opens a ligand-gated Ca^2+^ channel in the endoplasmic reticulum leading to an increase in cytosolic Ca^2+^. Ca^2+^ in the cytosol exerts its effects by binding to Ca^2+^-binding proteins.

**Figure 4:**
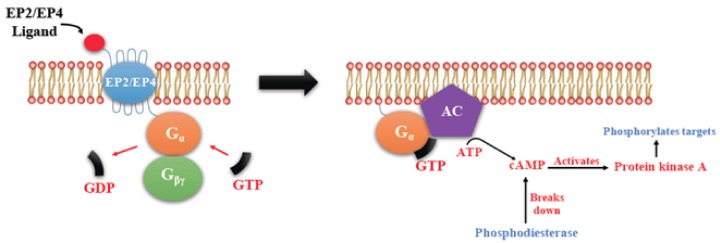
Shared pathway of EP2/EP4 mediated Signaling. There is activation of adenylyl cyclase (AC) leading to a rise in the second messenger cAMP in the cytosol that activates Protein kinase A (PKA). PKA in turn activates a transcription factor CREB (cAMP response element-binding protein).

**Figure 5:**
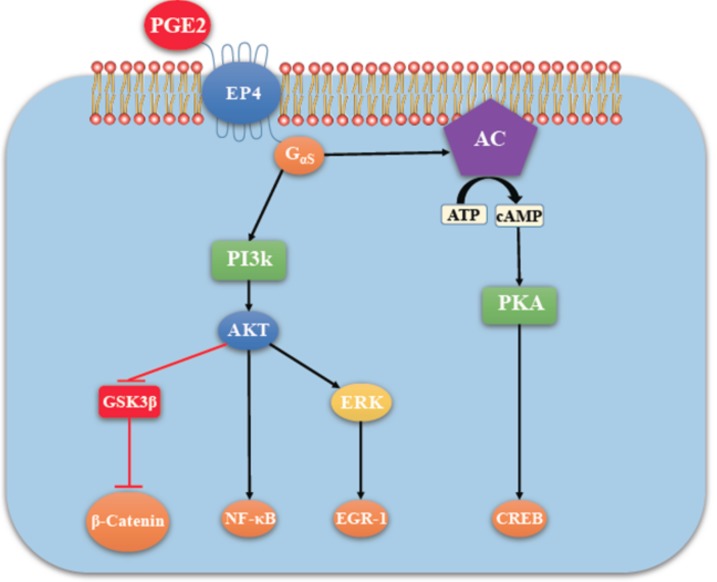
EP4 mediated signaling (in addition to PKA activation) not shared by EP2 (adapted with kind permission from O’callaghan, G.; Houston, A.; 2015; reference [13]). There is non-canonical activation of the PI3K-Akt and ERK pathways. Akt, also called protein kinase B (PKB) promotes cell survival by activating the transcription factor NF-κB. ERK is primarily a promoter of cell proliferation and migration. Cell proliferation depends on the ERK mediated activation of the transcription factor EGR-1.

**Figure 6:**
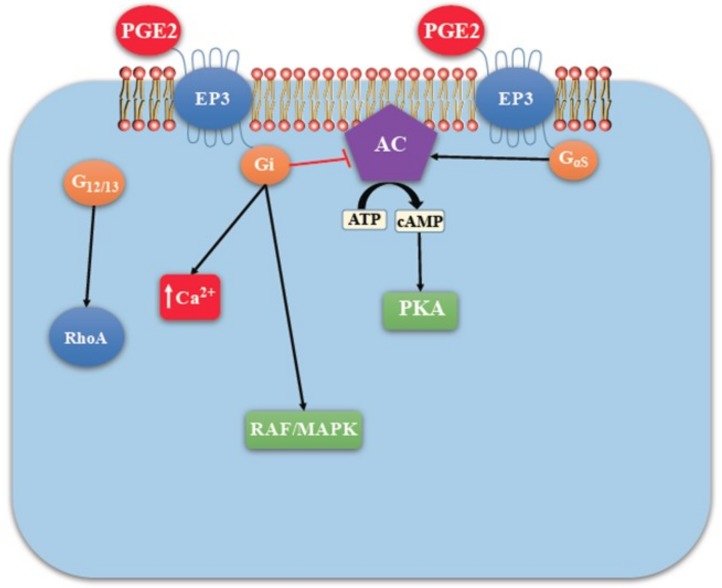
EP3 mediated signaling (adapted with kind permission from O’callaghan, G.; Houston, A.; 2015; reference [13]). EP3 has multiple isoforms, most of which are coupled with the inhibitory G-protein Gi that acts by inhibiting AC-cAMP-PKA pathway. Those coupled with Gs stimulate AC-cAMP-PKA pathway. Those coupled with G_12/13_ are involved in Rho family GTPase signaling utilized in cell migration by cytoskeleton remodeling.

PGE2-mediated intracellular signaling depend on its binding of target cells to one or more of the specific prostaglandin E receptors (EP1-4), which are coupled to different G-proteins. [10,11,12] (Figure 1). The activation or inactivation of G-proteins occurs as follows (Figure 2). When a ligand binds, the receptor activates the attached G-protein by causing the exchange of GTP for GDP. The activated G-protein then dissociates into an alpha (G-α) and a beta-gamma (G-β/γ) complex. GTP bound G-α is active, and can diffuse along the membrane surface to activate (and sometimes inhibit) target proteins, typically enzymes that generate second messengers. Similarly, the G-β/γ complex is also able to diffuse along the inner membrane surface and affect protein activity. Intrinsic GTPase activity is responsible for the inactivation of the G-protein. After GTP hydrolysis, GDP bound G-α will re-associate with a β/γ complex to form an inactive G-protein that can again associate with a receptor. After binding its cognate receptor, PGE2 is metabolized in a two-step process in which the prostaglandin is transported into the cytoplasm through a passive mechanism or actively by prostaglandin transporter (PGT), followed by the inactivation by 15-PGDH. Signaling mediated by the EP family depends on the coupled G-protein. As shown in Figure 3, EP1 couples with Gαq, activating phospholipase C (PLC) that cleaves PIP2, a membrane phospholipid, to generate second messengers, IP_3_, and diacylglycerol (DAG). IP_3_ is water soluble, diffusing through the cytosol to bind to and open a ligand-gated Ca^2+^ channel in the endoplasmic reticulum (or sarcoplasmic reticulum in muscle cells), leading to an increase in cytosolic Ca^2+^. Ca^2+^ in the cytosol exerts its effects by binding to Ca^2+^-binding proteins, such as calmodulin (Figure 3). EP2 and EP4 couple with Gαs, which activates the enzyme adenylyl cyclase (AC) and catalyzes the formation of the second messenger cyclic AMP (cAMP) (Figure 4). An activated AC can generate many molecules of cAMP within the cell to amplify the signal. The major effect of cAMP is to bind to and activate protein kinase A (PKA; also known as cAMP-dependent kinase). PKA then phosphorylates target proteins in the cell. cAMP is rapidly broken down by phosphodiesterases, limiting the length of the signal. Additionally, in contrast to EP2, EP4 also stimulates non-canonical pathways phosphatidylinositol 3 kinase (PI3K)/protein kinase B (PKB, also known as Akt) promoting cell survival, and extracellular regulated kinase (ERK), promoting migration and proliferation. The phosphorylation of EP4 receptor recruits β-arrestin-1, which in turn, activates c-Src to initiate the transactivation of the epidermal growth factor receptor (EGFR) and the subsequent downstream signaling through phosphatidyl inositol 3-kinase (PI3K) and Akt [13] (Figure 5). The activation of this signaling cascade has been proposed to regulate the migration and metastasis of colorectal carcinomas [14]. EP3 receptor mediated signaling (shown in Figure 6) depends on the coupling with several G-protein isoforms. Most are coupled with Gαi inhibiting cAMP-PKA; those coupled with Gαs stimulate cAMP-PKA; those coupled with Gα_12/13_ stimulate Rho family GTPases that are involved in cytoskeletal changes required for cellular migration. Exploitation of EP receptors as therapeutic targets by the development of selective agonists and antagonists has been elegantly reviewed [9,13,14]. Of all the receptors, the roles of EP4 receptor in health and disease have received much attention [15]. As documented below, COX-2 expressing breast cancers utilize the EP4 pathway for cancer cell survival and metastasis, making EP4 a targetable molecule for treating aggressive breast cancer patients. 

## 4. COX2/PGE2 Mediated Cancer Progression

Aberrant COX-2 expression promotes tumor initiation, progression, and metastasis in most epithelial cancers [16]. This has been shown by over-expression [17] and down regulation [18] of the *COX-2* gene, and the protective effects of the use of selective as well as non-selective COX-2 inhibitors from colorectal and mammary carcinogenesis [16,19,20,21,22,23]. COX-2 over-expression is a phenotype that is shared by aggressive cancers of the colon [24], lungs [25], pharynx and larynx [26], pancreas [27], and the breast [28]. Elevated COX-2 expression noted in 40–50% of breast cancer, marks poor prognosis [29], resulting from the high levels of PGE2 in the tumor microenvironment. Studies with murine breast cancer models have validated the roles of COX-2/PGE2 in tumor progression and the efficacy of COX-1/COX-2 inhibitors in halting tumor growth and metastasis [30,31,32,33].

### 4.1. EP Receptor Functions in Health and Disease Including Tumorigenesis: A Brief Overview

#### 4.1.1. Biological Functions of EP Receptors

The amino-acid sequence homology of different EP receptors and isoforms, tissue distribution, and biologicals functions of EP receptors have been elegantly reviewed by Sugimoto and Maruyama [12]. Following is a summary of the physiological and pathological roles of each EP receptor, identified from the use of individual EP receptor-deficient mice. EP1 mediates stress-related responses, including ACTH secretion, promotes chemical carcinogenesis, and mediates inflammation-induced thermal hyperalgesia. EP2 facilitates ovulation and fertilization, assists pain transmission by abolishing glycinergic inhibition, mediates joint inflammation in collagen-induced arthritis, suppresses dendritic cell differentiation, facilitates neutrophil recruitment by Granulocyte Colony Stimulating Factor (CSF) production, promotes amyloid-β formation in Alzheimer’s disease, mediates COX-2-induced mammary hyperplasia, and promotes intestinal polyp formation. The latter was shown by reduced polyp formation in polyp-prone APC delta-716 knock out mice. EP3 regulates duodenal secretion, mediates fever generation under pyrogenic conditions, suppresses type I allergy, mediates angiogenesis associated with tumor development and chronic inflammation, induces endotoxin-elicited enhanced pain perception, and mediates pain that is associated with virus infection. EP4 facilitates closure of ductus arteriosus at birth, induces osteogenesis, protects against inflammatory bowel disease, facilitates Langerhans cell migration and maturation, and mediates joint inflammation in collagen-induced arthritis.

#### 4.1.2. EP Receptors in Tumorigenesis

Callaghan and Houston [13] provided a comprehensive review of various EP receptors and their signaling pathways in tumorigenesis in various organs, concluding that multiple EP receptor pathways are involved, and that a combination of EP receptor antagonists may hold better promise in therapy than a single antagonist, depending on the cancer type. Reader et al. [34] provided a comprehensive up-to-date (2011) review of the literature reporting the roles of different EP receptors in normal mammary development, post-natal changes in mammary gland during pregnancy and lactation, mammary neoplasia, and malignant behavior in various tissues. The findings demonstrated that the EP receptors are expressed in a variety of malignant cells as well as the tumor stroma and that their roles in malignancy are tissue and cell-dependent; that some of the receptors are potential therapeutic targets. Multiple subcellular locations of the same receptor other than the plasma membrane, such the nucleus posed additional complexity in identifying receptor functions. Expression of EP receptors in the stroma and by host immune effector cells also appeared to play a role in tumor behavior. 

#### 4.1.3. EP Receptors in Colorectal Carcinogenesis

A large body of evidence suggests that the COX-2 plays a major role colorectal carcinogenesis [23,24]. This was shown to be due to PGE2-EP receptor signaling [35]. Numerous studies that were reviewed by Hull et al. [36] suggested that EP1, EP2, and EP4 receptors play promoting roles in the early stages of intestinal tumorigenesis. Genetic depletion of EP receptors implicated all of the EP receptors, in particular, EP4 in colonic carcinogenesis (reviewed in [37]). Using human colonic adenocarcinoma cell line HCA-7 as an in vitro model, it was shown that there was EP2/EP4 mediated cAMP/PKA pathway signaling, followed by biphasic activation of ERKs related to EP4 activation [37]. Chell et al. [38] reported that EP4 receptor protein expression was increased in colorectal cancers (100%), as well as adenomas (36%) when compared with normal colonic epithelium. EP4 expression was also higher in colorectal carcinoma cell lines, when compared with adenoma cell lines and increased with in vitro models of tumor progression. Both in vivo and in vitro data from this study suggest that increased EP4 receptor expression is important during colorectal carcinogenesis and that the EP4 may represent an important target for colorectal cancer prevention and treatment [38]. In support, it was shown that EP4 mediated signaling involving β-arrestin 1 is important in the metastatic progression of colorectal cancer [14].

#### 4.1.4. EP Receptors in Skin Carcinogenesis

Roles of EP receptors in skin carcinogenesis have been explored in several models. During the process of two-stage chemical carcinogenesis in the skin (treatments with DMBA, followed by TPA in mice deficient in EP3 receptor) Shoji et al. [39] reported that EP3-mediated signaling might contribute to the development of squamous cell carcinoma. Rundhaug et al. [40] reviewed the roles of COX-2/EP receptors in UV-induced skin carcinogenesis. They concluded that the combination of EP1, EP2, and EP4, but not EP3-mediated signaling was important for activating pathways that are responsible for UV induction of inflammation, proliferation, and tumorigenesis. They emphasized the role of EP4. For example, transgenic mice over-expressing EP4 under the control of the BK5 promoter (BK5.EP4 mice) when subjected to the two-stage DMBA/TPA protocol, developed many more tumors than wild-type mice. 

#### 4.1.5. EP Receptors in Carcinogenesis in the Mucosa of the Pharynx and the Esophagus

Studies of the roles of COX-2 and EP receptors in head and neck squamous cell carcinomas (HNSCC) revealed COX-2 over-expression and the expression of all EP receptors and that EP3 receptor may play a more prominent role in HNSCC cell growth promotion [41]. Lowery et al. [42] reviewed the roles of EP receptors in esophageal carcinogenesis. COX-2 over-expression was noted in Barrett’s esophagus (a pre-malignant lesion) and esophageal carcinomas, suggesting that these patients are candidates for chemo-preventive and/or therapeutic approaches. The authors compared the potential of traditional NSAIDs, selective COX-2 inhibitors, and EP antagonists as chemo-preventive and/or therapeutic agents.

#### 4.1.6. EP Receptors in Prostatic Carcinogenesis

The role of EP receptors in prostate cancer progression, as reviewed by Nithipatikom and Campbell [43], suggested the roles of EP2 and EP4 in tumor progression. In support, EP4 over-expression in prostatic carcinoma cells was associated with castration-resistant phenotype, and treatment with an EP4 antagonist demonstrated anti-invasive effects in vitro and halted bone metastasis in tumor-transplanted mice [44]. 

#### 4.1.7. EP Receptors in Urothelial Carcinogenesis

EP2/EP4 activation was shown to correlate with induction of urothelial cancer initiation and outgrowth, as well as bladder cancer progression and resistance to cisplatin, presumably via downregulating PTEN expression [45]. In bladder cancer cell lines, EP2/EP4 antagonists, and the COX-2 inhibitor celecoxib effectively inhibited cell viability and migration, as well as augmented PTEN expression. 

#### 4.1.8. EP Receptors in Non-Small Cell Lung Cancer

While non-small cell lung cancers (NSLC) exhibited high COX-2 expression, there were no distinct patterns of EP receptor expression in tumors, and EP receptor expression appeared to be modified epigenetically [46].

### 4.2. COX-2/PGE2 Mediated Breast Cancer Progression: Role of EP4 Receptor

**Figure 7:**
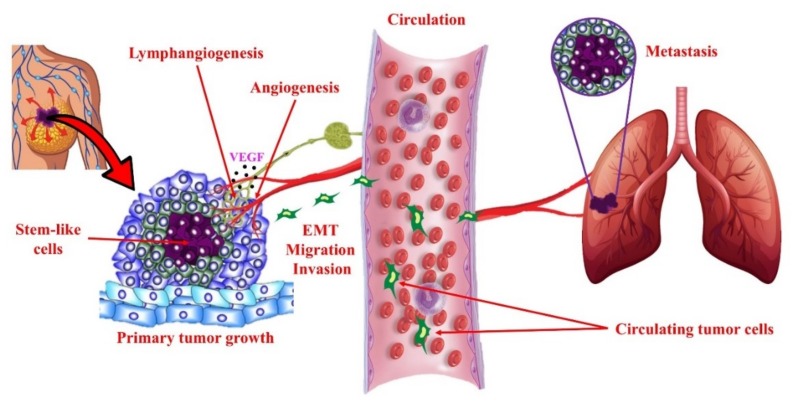
Schema of cellular events in tumor progression and metastasis. Primary tumor growth depends on proliferation of tumorigenic cells, some of which adopt a stem-like cell (SLC) phenotype under the influence of genetic and epigenetic (micro-environmental) mechanisms. Local tumor growth is dependent on angiogenesis (formation of new blood vessels), which also facilitates tumor cell egress into the circulation. In addition, many epithelial tumors undergo intra-tumoral and/or peri-tumoral lymphangiogenesis (formation of new lymphatic vessels) that helps tumor cells to migrate to lymph nodes and then enter circulation. Epithelial-mesenchymal transition (EMT) is a phenotypic change in epithelial tumor cells utilized for invasion and migration out of the local confines. These cellular events are stimulated in COX-2 expressing breast tumors by activation of EP4 on tumor cells and tumor-associated host cells (immune cells, endothelial cells), so that EP4 presents as a therapeutic target to block multiple cellular events in tumor progression.

Elevated COX-2 expression (noted in about half of breast cancer patients) signals poor prognosis [29], associated with large tumor size, high histological grade, negative hormone receptor status, high proliferation rate, ductal type histology, high p53 expression, HER-2 oncogene amplification, and axillary node involvement. We demonstrated that COX-2 over-expression leads to high endogenous PGE2 levels that promote breast cancer progression by multiple mechanisms: inactivation of host anti-tumor immune cells [30,31], enhanced cancer cell migration [47,48], invasiveness [47,49], tumor-associated angiogenesis [47] due to upregulation of angiogenic factors, and tumor-associated lymphangiogenesis [50,51,52,53] due to the upregulation of lymphangiogenic factors VEGF-C and -D. These events were primarily due to the activation of the PGE2 receptor EP4 on tumor and host cells, as listed below. EP4 activity on tumor cells promoted tumor cell migration, invasion, angiogenesis, and lymphangiogenesis [47,48,50,51,52,53]. The downstream signaling for EP4 activity in these events included cAMP/PKA pathway shared by EP2, and PI3K/Akt pathway unique for EP4. We further observed that in COX-2 expressing breast cancer cells, under inductive conditions, endogenous PGE2 upregulated iNOS by the activation of EP4 to promote invasive functions [49], which may be cGMP/PKG dependent. Other investigators reported that EP4 on host NK cells [54,55] and T cells [56] blocked their killer functions. We found that EP4 on tumor-associated macrophages [52] promoted their lymphangiogenic function by upregulating VEGF-C or -D. Similarly, EP4 activation on host lymphatic endothelial cells (LEC) promoted lymphangiogenesis, resulting from stimulated LEC proliferation, migration, and tube formation triggered by upregulation of VEGF-C or -D and VEGFR3 [53]. EP4 activation on host macrophages also promoted their immune-suppressor functions [57]. EP2/EP4 activity on dendritic cells blocked their antigen-presenting function [58]. Finally, we discovered that COX-2/EP4 activities also induced and sustained stem-like cell (SLC) phenotype in breast cancer cells in a syngeneic murine breast cancer model [52] and human breast cancer cells [59]. Similar findings have been reported in a different murine breast cancer model [60]. SLCs are a minor subpopulation of cells within tumors, which have an unlimited self-renewal capacity [61,62], and their activities are regulated by the microenvironment, indicating that they have a plastic phenotype. They resist conventional chemo/radiation therapies, frequently leading to recurrence of primary or metastatic tumors, necessitating the search for SLC-specific markers, and therapeutic targets [63,64]. We suggest that EP4 is a highly suitable therapeutic target to eliminate SLCs, and therefore impact breast cancer metastasis. A schema of the cascade of cellular events in breast cancer progression and metastasis, which can be blocked with EP4 antagonists, is presented in Figure 7.

### 4.3. Other Molecular Players Upstream or Downstream of EP4 that Promote Breast Cancer Progression

EP4 can serve as an intermediary in multiple mechanisms in breast cancer progression. Upregulation of the sphingosine 1-phosphate (S1P) 3 receptor in highly metastatic variants of a breast cancer cell line was shown to increase migration and invasion by the induction of PGE2 and EP2/EP4 activation [65]. Genome-wide DNA methylation and expression analysis of breast cancer cell line models of acquired estrogen resistance indicated that the EP4 receptor was upregulated by epigenetic mechanisms in demethylation resistant breast cancer cells [66]. In this study, EP4 was shown to be essential for estrogen-independent growth resulting from the activation of the ERα-cofactor CARM1. Thus EP4 is a justifiable target in endocrine therapy-resistant breast cancer. Furthermore, EP4 activation can also lead to transactivation of other pathways in breast cancer progression: (1) Transactivation of the intracellular signaling pathway of the epidermal growth factor receptor (EGFR). It was shown that the EGFR pathway activation promotes the formation of invadopodia in breast cancer cells, which increases their capacity to degrade the surrounding extracellular matrix [67]. Thus, EP4/EGFR cross talk is another mechanism for increased invasiveness in breast cancer cells. (2) The chemokine receptor CCR7 is another downstream molecule upregulated by EP4. It was shown that that EP2/EP4 mediated CCR7 upregulation enhanced the migration of breast cancer cells toward lymphatic endothelial cells and to promote lymphatic invasion [68]. CCR7 expression in COX-2-overexpressing tumors was significantly correlated with lymph node metastasis. Subsequent studies by this laboratory [69] revealed that CCR7 upregulation resulted from AKT-mediated phosphorylation and the activation of the transcription factor Sp1 in breast cancer cells. This signaling pathway is typical of EP4 activation, thus reinforcing the role of EP4.

### 4.4. Rationale for the Choice of EP4 as a Potential Therapeutic Target in Breast Cancer

While the intake of COX-2 inhibitors can reduce breast cancer risk and morbidity [21,70,71,72], their reported cardiovascular side effects [73,74] necessitated the search for alternative downstream target(s) that may spare vaso-protective functions of prostanoids. We suggest that EP4 represents as an ideal target for breast cancer to replace COX-2 inhibitors for three reasons: (i) the primary roles of EP4 in COX-2 mediated breast cancer progression listed above. (ii) EP4 is relatively redundant for many physiological functions shared by EP2 via PKA stimulation [10,11,12] (iii) vasoprotective actions of prostanoids were shown to be primarily mediated by IP and EP3 receptors, as suggested by findings in a variety of animal models of cardiac ischemia. For example, PGI2 has been reported as a cardio protective prostanoid, implicating IP-mediated action in hypoxia-induced pulmonary hypertension and intravascular thrombosis [75]. In support, using IP^−/−^ and TP^−/−^ mice, it was shown that IP but not TP receptor was cardio protective. PGI2, which was produced endogenously during cardiac ischemia/reperfusion, exerted a protective effect on cardiomyocytes independent of its effects on platelets and neutrophils [76]. Furthermore, PGE2 was shown to mediate cardio protective effects via EP3 receptor activation in myocardial ischemia models. Ischemic myocardial injury was attenuated in transgenic mice with cardio-specific over-expression of the EP3 receptor [77]. In support, structurally diverse EP3 agonists could reduce myocardial infarct size in rats. The therapeutic effect was mediated by PKC activation and the opening of KATP (ATP-sensitive K) channels [78]. However, one study also implicated EP4 receptor. An EP4-selective agonist EP4RAG attenuated myocardial dysfunction after infarction and reduced infarction size in a rat myocardial ischemia/reperfusion injury model. The effects of the EP4 agonist appeared to be indirect by suppressing monocyte chemo-attractant protein-1 (MCP-1) and the infiltration of inflammatory cells, especially macrophages [79]. While no human data are available on whether EP4 antagonists can cause cardiovascular toxicity, an EP4 antagonist AAT-007 used in phase 1/2 trials in >800 human arthritis patients was well tolerated in pharmacologically effective doses (300 mg orally twice daily), with no evidence of dose-limiting toxicity (Dr. Yukinori Take, Ask/At, Japan, personal communication, cited with permission).

### 4.5. Functional Roles of COX-2 in the Absence or Presence of HER-2 in Breast Cancer

Human epidermal growth factor receptor (HER) 2 expressed by approximately 20% breast cancer patients is another major driver of breast cancer progression. HER-2 is often co-expressed with COX-2 in human breast cancer [80], although the reverse in not true. Interestingly, most HER-2 actions e.g., upregulation of aromatase [81], angiogenesis [82], lymphangiogenesis [80], and anti-apoptotic action [83] were shown to be intermediated by COX-2. To define the functional roles of COX-2 in the absence or presence of HER-2, we stably transfected *COX-2* gene into MCF-7 (COX-2-, HER-2-) and SKBR-3 (COX-2-, HER-2-high) human breast cancer cell lines [59]. Ectopic COX-2 over-expression in MCF-7 and SKBR-3 cell lines resulted in: increased migration/invasion/proliferation, epithelial-mesenchymal transition (EMT), elevated SLCs (spheroid forming ability in vitro), increased ALDH activity- a recognized SLC marker [84] and the co-localization of COX-2 with numerous SLC markers (ALDH1A, CD44, β-Catenin, NANOG, OCT3/4, and SOX-2) in spheroids. These changes were reversed with COX-2-inhibitor or EP4-antagonists (EP4A), indicating the dependence on COX-2/EP4 activities. COX-2 over-expression or EP4-agonist treatments of COX-2-low cells caused up-regulation of stem cell related *NOTCH/WNT* genes, blocked with PI3K/AKT inhibitors. NOTCH/WNT inhibitors also blocked COX-2/EP4 induced SLC induction. Microarray analysis showed an up-regulation of numerous SLC-regulatory and EMT-associated genes. MCF-7-COX-2 cells showed increased mammary tumorigenicity and spontaneous multi-organ metastases in NOD/SCID/IL-2Rγ-null mice for successive generations with limiting cell inocula [59], which is a rigorous test for testing SLC in vivo [85]. Conversely, lung colonization was abrogated with EP4 knockdown or EP4 antagonist treatment of the cells. Orthotopic mammary tumors that were grown with MCF-7-COX-2 cells (as compared to control cells) showed an up-regulation of angiogenic/lymphangiogenic factors VEGF-A/C/D, Vimentin (mesenchymal marker), and phospho-AKT (an EP4 signaling marker), down-regulation of epithelial marker E-Cadherin and an enrichment of SLC marker positive and spheroid forming cells. Findings in primary human breast cancer tissues were supportive of the findings in mice, as noted above. Expression of *COX-2*, *EP4*, and *ALDH1A* mRNA in these tissues were highly correlated with one other, more marked in progressive stage of disease. In situ immunostaining of the tissues revealed the co-localization of SLC markers with COX-2, supporting SLC induction by COX-2. Finally, high *COX-2*/*EP4* mRNA expression was linked with reduced survival [59]. Inflammatory breast cancers (IBC), although uncommon, also express high levels of COX-2. Using IBC cell lines, SUM149, and SUM190, it was shown that the EP4 antagonist GW627368X and shRNA-mediated EP4 knockdown blocked invasive capacity of IBC cells [86]. These preclinical and clinical data strongly suggest that EP4 represents a novel therapeutic target to inhibit tumor growth, metastasis, and eradicate SLCs in human breast cancer. This contention was fully validated by us in mouse breast cancer models [51,52] with two EP4 antagonists (ONO-AE3-208, ONO pharma, Japan; and, RQ-15986, currently renamed as AAT 007, Ask/At Pharma, Japan). Treating mice bearing syngeneic COX-2 expressing, highly metastatic C3L5 mammary tumors with EP4A at non-toxic doses inhibited tumor growth, spontaneous metastasis, and eradicated SLCs in residual tumors [51,52]. Similar findings have been reported in another murine breast cancer model [60] with AAT 007. Similarly, therapeutic efficacy of ONO-AE3-208 in halting bone metastasis was reported in a castration-resistant prostate cancer model [44].

### 4.6. SLC-Linked MicroRNAs Induced by COX-2/EP4 Activity as Breast Cancer Biomarkers

**Figure 8:**
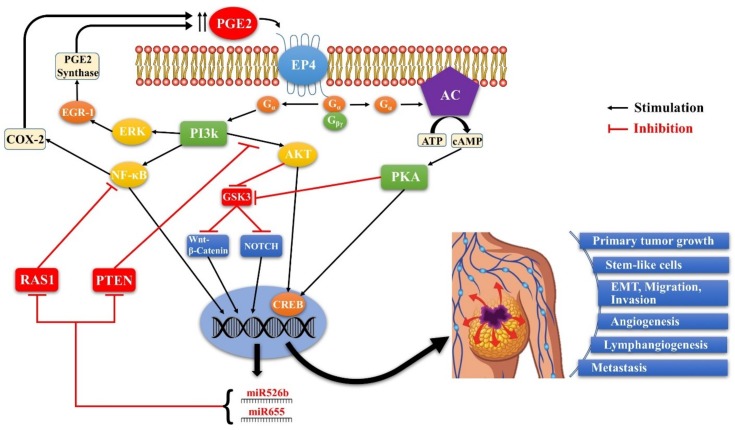
Schema of EP4 mediated signaling pathways in COX-2 expressing breast cancer. Aberrant COX- 2 activity leads to tumor progression and metastasis by utilizing multiple signaling pathways in which EP4 activation plays a pivotal role, and two COX-2/EP4 upregulated miRNAs (miR526b and miR655) are important partners. EP4 activation (like EP2) results in cAMP-dependent PKA activation leading to phosphorylation of the transcription factor CREB. PKA also upregulates WNT/β-catenin and NOTCH pathways by inhibiting GSK3. Furthermore, unlike EP2, EP4 also utilizes the non-canonical PI3K/AkT and ERK signaling pathways, respectively promoting cell survival and migration/ proliferation. COX-2 upregulates the miRNAs miR526b and miR655 via EP4 mediated PI3K/Akt activation and WNT/β-catenin/NOTCH pathways. While COX-2 induces these miRNAs, the miRNAs, in turn, upregulated COX-2. We suggest that these occur via upregulation of NF-κB, which is a well-known upregulator of COX-2 under certain conditions. Predicted targets of these miRNAs include NF-κB repressor genes. Thus there appears to exist a positive feedback loop for COX-2/EP4/NF-κB/miRNA/COX-2-mediated SLC perpetuation.

There are very few reliable blood biomarkers for breast cancer that are useful to monitor the disease. Levels of specific miRNAs in blood plasma remain as a newer family of cancer biomarkers. MiRNAs are single stranded non-coding RNAs (20–24 nucleotides) that down-regulate specific genes at the post-transcriptional level. There are 1917 sequences in the human miRNA registry [87], with some proposed as cancer biomarkers [88,89], which can be detected in body fluids [90] due to exosome-mediated release and transit in the blood. Recently, levels of a panel of seven candidate miRNAs were measured in tissue and blood specimens of 148 patients with minimally invasive breast cancer and 44 age-matched and disease free control individuals [91]. The authors found increased levels of blood miR-195 in breast cancer patients, which decreased to control levels following curative tumor resection. The circulating miRNAs correlated with certain clinic-pathological variables, namely nodal status and estrogen receptor status. We conducted differential gene and miRNA expression micro arrays using control MCF-7-Mock-tranfected vs. MCF-7-COX-2 transfected cell lines, which identified the downregulation of six miRNAs and an upregulation of two miRNAs (miR-655 and miR-526b) by COX-2. Both COX-2 upregulated miRNAs were also inducible by EP4 activation by exposing MCF-7 cells to selective EP4 agonists. Both miRNAs were shown to be highly oncogenic and SLC-linked [92,93]. Expression of both miRNAs positively correlated with COX-2 in genetically disparate breast cancer cell lines and increased in all cell lines when grown as spheroids. Spheroid assay is a vitro surrogate for measuring the self-renewal of stem-like cells (SLC), indicating the link of both miRNAs with SLC activity. Ectopic miR-526b or miR-655 over-expression in MCF7 and SKBR3 cells resulted in increased proliferation, migration, invasion, spheroid formation, and Epithelial to Mesenchymal transition (EMT). Conversely, knocking down either miRNA in aggressive MCF7-COX-2 and SKBR3-COX-2 cells reverted these phenotypes. MCF7-miR526b and MCF7-miR655 cells displayed upregulated *NOTCH*/*WNT* genes; both pathway inhibitors abrogated miRNA-induced spheroid formation, linking both miRNAs with SLC-related pathways. The expression of both miRNAs was dependent on EP4 activity and EP4 downstream signaling pathways PI3K/AKT, ERK, and NF-κB. Interestingly, while both miRNAs were upregulated in ectopic COX-2 expressing cells, ectopic miRNA overexpressing cells also upregulated COX-2. We suggest that this is due to the targeting of NF-κB repressor genes by both miRNAs. Thus there is a positive feedback loop for COX-2/EP4/NF-κB/miRNA/COX-2-mediated SLC perpetuation [92,93]. MiR-655 expression also led to TGFβ resistance for Smad3 phosphorylation [93]. Tail vein injection of ectopic miR-526b or miR-655 over-expressing MCF7 and SKBR3 cells into NOD/SCID/GUSB-null mice revealed increased lung colony growth and micro-metastases to other organs. Expression of both miRNAs was strongly correlated with each other in human breast cancer tissues, was higher than in non-tumor tissues, and was associated with reduced patient survival [92,93]. Thus, they could serve as prognostic breast cancer biomarkers for monitoring SLC-reduction during therapies. In support, our preliminary data (not presented) reveal that miR-655 levels are significantly higher in the plasma of patients bearing breast cancer than in patients with benign breast tumors. In summary, we found that aberrant COX-2 activity in human breast cancer leads to tumor progression and metastasis by utilizing multiple signaling pathways in which EP4 activation plays a pivotal role, and two COX-2/EP4 upregulated miRNAs are important partners (schema presented in Figure 8).

### 4.7. Triple-Negative Breast Cancers (TNBC) Are Mostly COX2 Expressing

TNBC represents the most deadly type of breast cancer, which resist cytotoxic therapies. Gene expression patterns that were gleaned from publicly available databases suggested that TNBCs expressed multiple drug resistance-associated protein (MRP) 4 (an active PGE2 exporter), low PGT (a PGE2 importer), and low 15-PGDH (a PGE2 catabolizer) [94]. They collectively favored the maintenance of high levels of PGE in the tumor microenvironment that may contribute to poor prognosis. Two studies reveal that TNBCs are mostly COX-2 expressing. In an earlier study [80], which was designed to identify the roles of COX-2 and HER2 in VEGF-C expression and lymphangiogenesis, we used 65 human breast cancer tissue samples and multiple human breast cancer cell line that was genetically manipulated for COX-2 and HER2 expression. We concluded that COX-2 was a primary driver of lymphangiogenesis, and the role of HER2, if any, was intermediated by COX-2. Interestingly, most HER-2 expressing tumors identified by immunohistology were also COX-2 positive. In addition, all of the 23 tumors identified as TNBC in this study also expressed COX-2 (unpublished). Similar findings have been reported by another laboratory [95] in 35 primary TNBC showing that COX-2 is over-expressed in these tumors (*p* < 0.009). Since we found that most COX-2 mediated mechanisms in breast cancer progression result from EP4 activation, we suggest that TNBCs will respond to EP4 antagonist (EP4A) therapy and miR655 could be used as a plasma biomarker for therapeutic monitoring in TNBC patients. Our future goal is to use EP4A as an adjunct in metastatic TNBC patients. However, it is currently unknown whether EP4A as a single agent will provide any benefit, as observed in our syngeneic murine breast cancer models [51,52]. We suggest that a combination therapy with other agents, such as immune checkpoint inhibitors holds a greater promise. 

### 4.8. Proposed Combination of an EP4 Antagonist with an Immune Checkpoint Inhibitor for Treating TNBC

Programmed cell death (PD)-1 is a checkpoint protein on T cells that normally acts as an “off switch” preventing them from attacking other cells in the body. This is mediated by binding of PD-1 to its ligand PD-L1 produced by other cells. Some cancer cells produce large amounts of PD-L1, which helps them to evade immune attack by T cells even if they can recognize tumor-associated antigen. This appears to be a defense mechanism hijacked by many solid tumors, leading to a recent renewal of interest in immunotherapy with immune checkpoint (PD-1, PD-L1) inhibitors. They have shown promise in multiple solid tumors in the human [96,97,98,99]. A recent study [100] reported a heterogeneous PD-L1 expression in primary breast cancer tissues, which are generally associated with the presence of tumor-infiltrating lymphocytes and poor-prognostic features such as high grade, and aggressive molecular subtypes (TNBC, basal, HER2^+^). Early phase clinical trials using PD-1 or PD-L1 inhibitors alone or in combination revealed objective tumor responses and durable long-term disease control in heavily pre-treated patients, notably in the TNBC [100]. We believe that a combination therapy using a PD-1 or PD-L1 inhibitor with an EP4 antagonist will improve the therapeutic efficacy of either drug. As summarized earlier, EP4 antagonists abrogate multiple mechanisms in breast cancer progression by binding to EP4 on multiple cell classes, like tumor cells, host immune cells, and endothelial cells. EP4 on tumor cells promote tumor cell migration, invasiveness, EMT, stem cell activity, angiogenesis (by upregulating VEGF-A), and lymphangiogenesis (by upregulating VEGF-C/D); EP4 on lymphatic endothelial cells promote lymphangiogenesis by upregulating VEGF-C/VEGFR3. Furthermore, PGE2 meditated inactivation of host antitumor immunity was shown to be due to EP4 binding on multiple immune cell classes: NK cells, T cells, macrophages, and dendritic cells. EP4 antagonists were shown to be highly effective in abrogating all of these events in animal models leading to tumor cell killing [51,52,60]. As outlined earlier, immune checkpoint inhibitors work via different non-overlapping mechanisms. Thus, it is expected that a combination of the two should cast the net far wider to block multiple tumor and host cell mediated pathways, leading to a synergistic action. Indeed a synergistic action on tumor regression and animal survival was shown with an EP4 antagonist in combination of either of two checkpoint inhibitors, anti-CTLA4 and anti-PD-1 antibodies, in murine colon and breast cancer models [101]. 

### 4.9. EP4 Antagonist in the Breast Cancer Clinic

While numerous EP4 antagonists have been tested for efficacy in animal breast cancer [51,52,55,60], prostate cancer [44], and melanoma [102] models, none of them has so far undergone human trial in cancer patients. A phase 2 human trial with the EP4 antagonist AAT 007 (AskAt, Nagoya, Japan) was recently registered by Dr. Martin Edelman at the University of Maryland (currently at FOX Chase Cancer Centre) in advanced solid tumors, including prostate, breast, or non-small cell lung cancer (Clinical Trials.gov Identifier: NCT02538432, last update posted on 6 June 2017). The trial is designed to test (a) whether the administration of the study drug AAT 007 can decrease circulating tumor cells or myeloid-derived suppressor cells; and (b) whether the drug may improve an outcome on its own in these solid tumors or when combined with a cytotoxic drug gemcitabine in patients with prostate or lung cancer, if the disease worsened with AAT 007 alone. No patient registration or outcome has yet been reported. A newer and more potent EP4 antagonist, named as AAT-008, having significantly improved pharmacological profiles and bio-availability has recently been reported by Okumura et al. [103] at the Ask/At. This compound has yet to be tested in the clinic.
